# Main Sources, Socio-Demographic and Anthropometric Correlates of Salt Intake in Austria

**DOI:** 10.3390/nu10030311

**Published:** 2018-03-06

**Authors:** Verena Hasenegger, Petra Rust, Jürgen König, Anna Elisabeth Purtscher, Judith Erler, Cem Ekmekcioglu

**Affiliations:** 1 Department of Nutritional Sciences, University of Vienna, Althanstrasse 14, 1090 Vienna, Austria; verena.hasenegger@univie.ac.at (V.H.); juergen.koenig@univie.ac.at (J.K.); 2 Department of Environmental Health, Center for Public Health, Medical University of Vienna, Kinderspitalgasse 15, 1090 Vienna, Austria; cem.ekmekcioglu@meduniwien.ac.at; 3 Health University of Applied Sciences, Tyrol, Bachelor Programm Dietetics, Innrain 98, 6020 Innsbruck, Austria; anna-elisabeth.purtscher@fhg-tirol.ac.at (A.E.P.); Judith.erler@fhg-tirol.ac.at (J.E.)

**Keywords:** salt intake, socio-demographic correlates, sources of salt, anthropometry

## Abstract

Excessive salt intake is known to increase blood pressure and cardiovascular risk. Nevertheless, salt intake exceeds the recommendations in most countries. To face this problem, it is important to identify high consumers as well as the main contributors of salt intake. Overall, data of 2018 adults between 18 and 64 years were analysed to determine the main sources, socio-demographic and anthropometric correlates of salt intake. Dietary intake was assessed from 24-h-recalls, information on socio-demographic characteristics was obtained using a questionnaire and anthropometric data were measured. Salt intake was significantly higher in males than in females. There was a significant positive association between salt intake and body mass index. No significant differences in salt intake were observed for other variables including affluence, educational level, smoking status and physical activity. The main contributor to salt intake were condiments including table salt (32.6%), followed by cereals and cereal products (27.0%), meat and meat products (16.1%) and dairy products (14.0%). These results highlight that specific population groups need to be targeted by public health initiatives and that a reduction in salt intake can only be achieved in tandem with the food producers by the reduction of salt in processed foods.

## 1. Introduction

It is well established that high dietary salt (sodium chloride) intake is associated with raised blood pressure (BP) and subsequently with adverse effects on cardiovascular health [1]. In detail, excessive salt consumption is related to coronary heart disease (CHD), stroke and renal disease [2,3,4,5,6], which are leading causes of death in Western countries. For example, complications of hypertension account for 9.4 million deaths every year worldwide [7].

Furthermore, there is also increasing evidence, that high dietary salt intake is indirectly associated with obesity through the relatively high fat and energy content of salty foods and the fact, that salty foods are more palatable and encourage people to consume greater quantities of these foods [8,9]. Besides, high dietary salt intake or preference for salty food is discussed to be linked to stomach cancer [10,11].

On the other hand, there is concern from few studies, that dietary salt restriction might also lead to adverse health effects by influencing blood lipids and insulin resistance [12].

To address these issues, the World Health Organization (WHO) recommends a reduction of sodium intake to <2 g/day (<5 g salt/day) [13]. According to the recommendations of the German Nutrition Society, the Austrian Nutrition Society and the Swiss Society of Nutrition salt intake should be reduced to <6 g/day, which corresponds to a sodium intake of <2.4 g/day [14]. In contrast to the current recommendations, the Prospective Urban Rural Epidemiology (PURE) study—one of the largest studies which observed the relationship of salt intake and human health—demonstrated a J-curve relationship between salt intake and adverse clinical outcomes. A sodium intake between 3 g per day (7.5 g salt/day) and 6 g per day (15 g salt/day) was associated with a lower risk of death and cardiovascular events than a higher or lower level of intake and raised the question if there is a need to re-evaluate existing recommendations [15]. However, this study had limitations like the inclusion of sick persons and suboptimal methods in the assessment of sodium intake.

The average current dietary salt intake is between 9 and 12 g per day in Western countries as well as in many non-industrialized countries. The dietary salt intake among adults in most European countries ranges from 7 to 13 g per day [16]. Only 10 to 12% of sodium occurs naturally in foods. In a typical Western diet, the salt content of processed and restaurant foods contribute three-quarters or more of sodium intake. The term “processed foods” covers all foods that have undergone manufacturing methods including not only convenience foods but also products like bread, cheese and meat products [17]. In non-industrialized countries, the main contributors of daily salt intake (up to 75%) is salt added during preparation, at the table at home or condiments (e.g., soy sauce, fish sauce) used for the seasoning of foods in countries such as China [18,19,20,21].

For the assessment of salt intake, the 24-h urinary sodium excretion method is considered as a gold standard [22] whereas the assessment in nutrition surveys often underestimates sodium intake. According to literature underestimation is around 29% to 41% [23] due to underreporting, difficulties in quantifying salt content of processed foods and discretionary salt use (in home cooking or at the table) [19,20]. Nevertheless, the assessment of dietary salt intake in nutrition surveys enables the identification of main sources of sodium intake, which is inevitable for public health interventions. Furthermore, it allows the linking of salt intake with dietary patterns or the intake of other nutrients [24]. Other types of studies which can be used to identify main sources of salt are for example the use of food composition and sales data [25].

There is little data available about socio-demographic correlates of salt intake to identify the typical high salt consumer. Additionally, it is crucial to identify the major sources of sodium to formulate effective public health and salt reduction strategies. Therefore, we investigated sodium intake in regard to socio-demographic correlates and identified major dietary sources of sodium intake in the Austrian adult population. Furthermore, predictors of sodium intake, like the body mass index, were studied.

## 2. Materials and Methods

### 2.1. Study Design

Data were collected within the Austrian Nutrition Survey 2014/2016, a regularly conducted representative cross-sectional survey to monitor food consumption, anthropometric characteristics and other health determinants such as physical activity and smoking behaviour in the Austrian population. The main target population was defined as adults living in private households in Austria aged between 18 and 64 years. Institutionalized subjects were excluded from the survey. Subjects were recruited using a multistage cluster sampling with a representative sample of Austrian companies as primary sampling units for people in paid work and different organizations such as the Austrian Public Employment Service (AMS) for unemployed population groups. Within the clusters, subjects were randomly selected and stratified according to sex and age. Therefore, the sample was representative for the adult Austrian population. The sample was composed of 2129 Austrian adults. Fieldwork was done between July 2014 and June 2016 to capture seasonal variation. Written, informed consent was obtained from all participants. The survey was approved by the Ethical Committee of the University of Vienna (reference number: 00284).

### 2.2. Anthropometric Measurements

Participants’ body heights were measured to the nearest 0.1 cm with a calibrated stadiometer (SECA 214 and SECA 217, SECA Vogel & Halke, Hamburg, Germany) in upright position with their heels standing together and body weights were measured to the nearest 0.1 kg on a calibrated digital scale (SECA BELLA 840 and SECA 877 Flachwaage, SECA Vogel & Halke, Hamburg, Germany) with participants being lightly dressed and without shoes. To correct for clothes 1 kg was subtracted from the measured body weights. Body mass index (BMI) was calculated by dividing each subject’s body weight in kg by the squared body height in m^2^. BMI was classified according to the World Health Organization [26], which defines underweight as BMI < 18.5 kg/m^2^, normal weight as BMI 18.50–24.99 kg/m^2^, overweight as BMI 25.00–29.99 kg/m^2^ and obesity as BMI ≥ 30.0 kg/m^2^.

Waist circumference was measured to the nearest 0.1 cm with an ergonomic measuring tape (SECA 201 and SECA 203, SECA Vogel & Halke, Hamburg, Germany) at the abdomen at the level of the iliac crest, after expiration [27].

### 2.3. Sociodemographic Characteristics

Information on level of education, affluence (Family Affluence Scale, [28]), physical activity (Global Physical Activity Questionnaire, [29]) and smoking status was obtained using a self-administered, web-based questionnaire.

### 2.4. Dietary Assessment

Dietary intake was assessed from repeated 24-h-recalls on two non-consecutive days using the software GloboDiet. GloboDiet was developed by the International Agency for Research on Cancer (IARC) within the European Investigation into Cancer and Nutrition Study (EPIC-Study) to carry out standardized 24-h-recalls [30]. The software was already used in numerous studies and validated within the EVCOVAL-Project [31,32,33]. Therefore, GloboDiet is one of the few dietary assessment tools which allow comparisons of dietary intake data across Europe. Currently, there are specific versions of GloboDiet in 18 European countries which are used in numerous national studies [34]. Within the Austrian Nutrition Survey 2014/2016 we adopted the software to local situations.

The reported foods collected during the interviews were linked to the German food composition database Bundeslebensmittelschlüssel 3.02. [35]. The conversion from dietary sodium intake to salt intake was made by multiplying the sodium value by 2.54. To identify under- or overreporters, basal metabolic rate (BMR) was calculated for each participant according to Schofield [36], taking into account sex, age, body height and body weight. Persons with a ratio of energy intake to estimated basal metabolic rate (BMR) lower than 0.788 or over 2.488 were excluded from the analysis according to Goldberg et al. [37] and Black [38].

### 2.5. Dietary Patterns

Diet of the participants was categorized using the Modified Mediterranean Diet Score (MMDS) [39]. Our intention was not to determine whether the eating habits of the participants were “healthy” or “unhealthy” but to classify the study population according to its nutritional habits.

A value of one was assigned for an intake above the sex- and cohort-specific median (g/1000 kcal/day) (Table 1) of five components considered as beneficial to health (vegetables including potatoes, fruits and nuts, legumes, cereals and fish). For an intake below or equal the median a value of zero was assigned. For two components presumed to be detrimental in case of high intake (meat and dairy products) a value of one was assigned for an intake below or equal the sex- and cohort-specific median (g/1000 kcal/day), otherwise a value of zero was given. As the traditional Mediterranean diet al.so includes a moderate consumption of alcohol [40], a value of one was assigned for an intake of ethanol to men who consumed between 10 and 50 g/day (4 and 20 g/1000 kcal/day adjusted for an energy intake of 2500 kcal/day) and to women who consumed between 5 and 25 g/day (2.5 and 12.5 g/1000 kcal/day adjusted for an energy intake of 2000 kcal/day), otherwise a value of zero was given. The Mediterranean diet pattern also includes the ratio of monounsaturated to saturated lipids [41]. We decided to choose the MMDS which includes not only monounsaturated but also polyunsaturated lipids in the numerator of the ratio, because in non-Mediterranean countries polyunsaturated lipids are the principle unsaturated lipids in the diet [39]. A value of one was assigned for a ratio above the sex- and cohort-specific median (g/1000 kcal/day) and a value of zero for an intake below or equal the median. Thus, the total MMDS ranged from zero (minimal adherence) to nine (maximal adherence). Participants were categorized in three groups: diet score 0–3 (low adherence), diet score 4–5 (medium adherence) and diet score 6–9 (high adherence).

### 2.6. Statistical Analysis

Results were expressed as median and interquartile range (IQ range) for continuous data and as percentage and 95% confidence interval (95% CI) for categorical data unless specified otherwise.

Data were tested for normal distribution using the Kolmogorov-Smirnov test. As the data on salt intake were left-skewed distributed, further analyses were carried out using non-parametric tests (Mann-Whitney-U test and Kruskal-Wallis test) to study differences between subgroups. Comparisons of metric data were done with bivariate correlations. In addition, a generalized linear model was used to study the effect of potential predictors on salt intake. The significance level was set at *p* < 0.05 for all analyses.

All statistical analyses were conducted using the statistics programme SPSS 22.0 (IBM, Armonk, NY, USA).

## 3. Results

Sample characteristics are presented in Table 2. A total of 111 misreporters were identified according to the afore-described definition and therefore excluded from the analysis leaving 2018 datasets for evaluation. Median age of participants was 38.0 (IQ range: 20.0) years, with females accounting for the majority (63.5%) of the sample.

### 3.1. Dietary Salt Intake

Only few data of the actual dietary salt intake are available in Austria. In 2010/2012 median dietary salt intake was 6.3 g/day when urinary sodium excretion was measured in spot urine and extrapolated to 24-h urine [44]. In the present study, median dietary salt intake was 5.0 (IQ range: 3.1) g/day with significantly (*p* < 0.001) higher consumption in males (6.1 (IQ range: 3.4) g/day) than in females (4.6 (IQ range: 2.7) g/day). Taking into account that sodium intake estimated by nutrition surveys may be underestimated by an average of 29% to 41% [23], the actual intake is very likely to be higher. The aim was not to assess the accurate intake quantity but to identify the main sources of salt intake.

No significant differences in dietary salt intake were observed between age groups (19–24 years (13.4% of participants): 5.0 (IQ range: 3.1) g/day), 25–50 years (66.1% of participants): 5.0 (IQ range: 3.1) g/day, 51–64 years (20.5% of participants): 5.2 (IQ range: 3.3) g/day).

### 3.2. Socio-Demographic Correlates of Dietary Salt Intake

Table 3 presents the socio-demographic characteristics of the participants and their daily dietary salt intake.

There was a significant positive association between dietary salt intake and body weight (r = 0.186, *p* = 0.001), body height (r = 0.241, *p* = 0.001), body mass index (r = 0.082, *p* = 0.001) and waist circumference (r = 0.153, *p* = 0.001).

Dietary salt intake was reported to be significantly lower (z = −3.640, *p* = 0.002) among normal weight (4.91 (IQ range: 2.89) g/day) compared to obese persons (5.80 (IQ range: 3.76) g/day).

Persons with a medium adherence (z = 2.734, *p* = 0.019) and those with a high adherence to the modified Mediterranean Diet (z = 2.813, *p* = 0.015) showed significantly lower dietary salt intakes than persons with a low adherence to the modified Mediterranean Diet.

However, no significant differences in dietary salt intake were observed for other variables including affluence, educational level, smoking status and physical activity.

### 3.3. Contribution of Foods to Dietary Salt Intake

**Figure 1:**
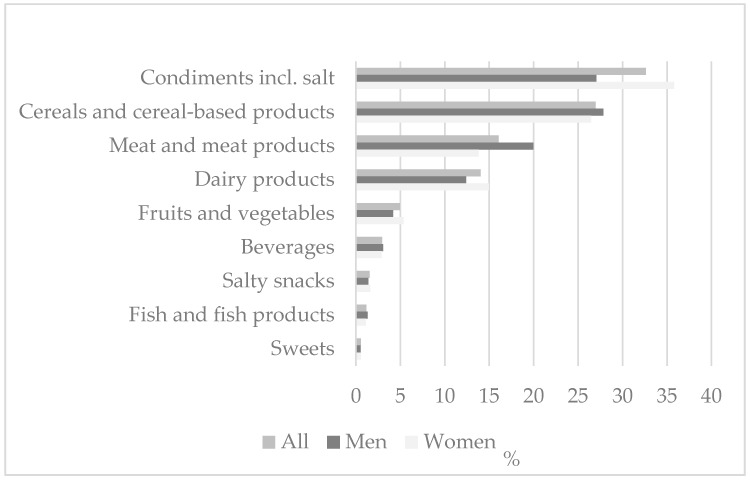
Contribution of different food groups to total dietary salt intake, all participants and by gender.

Figure 1 shows the contribution of different food groups to the total dietary salt intake. Cereals and cereal-based products accounted for 1.3 (IQ range: 1.2) g/day or 27.0% of the total dietary salt intake, with bread and bakery products alone contributing 26.1% to the total dietary salt intake (1.2 (IQ range: 1.2) g/day). Meat and meat products contributed 16.1% of salt in the diet (0.6 (IQ range: 1.3) g/day) and dairy products 14.0% (0.6 (IQ range: 0.7) g/day). Within these food groups meat products (0.5 (IQ range: 1.3) g/day–14.1%) and cheese (0.3 (IQ range: 0.6) g/day–8.4%) contributed the most to the total dietary salt intake.

Condiments including salt itself and condiments used in ready-to-eat meals accounted for 1.3 (IQ range: 1.5) g/day or 32.6% of the total dietary salt intake. Discretionary salt use (in home cooking or at the dining table) alone contributed 22.4% to the total dietary salt intake (1.0 (IQ range: 1.2) g/day). Therefore, the majority of consumed salt was from processed foods. The rest of dietary salt intake is attributable to fruits, vegetables and legumes, fish and fish products, beverages, salty snacks and sweets (11.2%).

### 3.4. Contribution of Food Groups to Differences in Dietary Salt Intake

**Figure 2:**
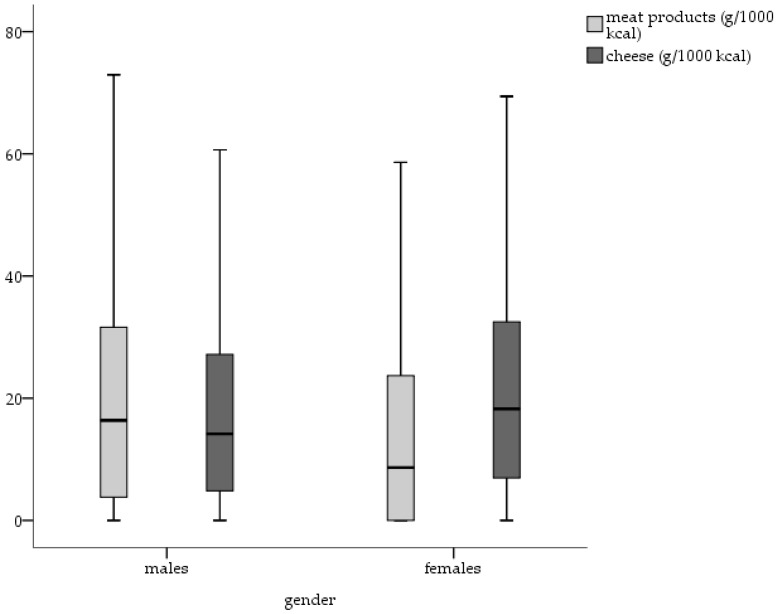
Daily intake of meat products (g/1000 kcal) and cheese (g/1000 kcal) by gender.

The intake of meat products was significantly higher in males than in females (U = 378,028, *p* = 0.001), even after adjusting for energy intake. In contrast, females showed a significantly higher intake of cheese (U = 518,427, *p* = 0.01) (Figure 2) and fruits, vegetables and legumes (U = 539,839, *p* = 0.01). No significant gender differences were found regarding the intake of bread and bakery products.

**Figure 3:**
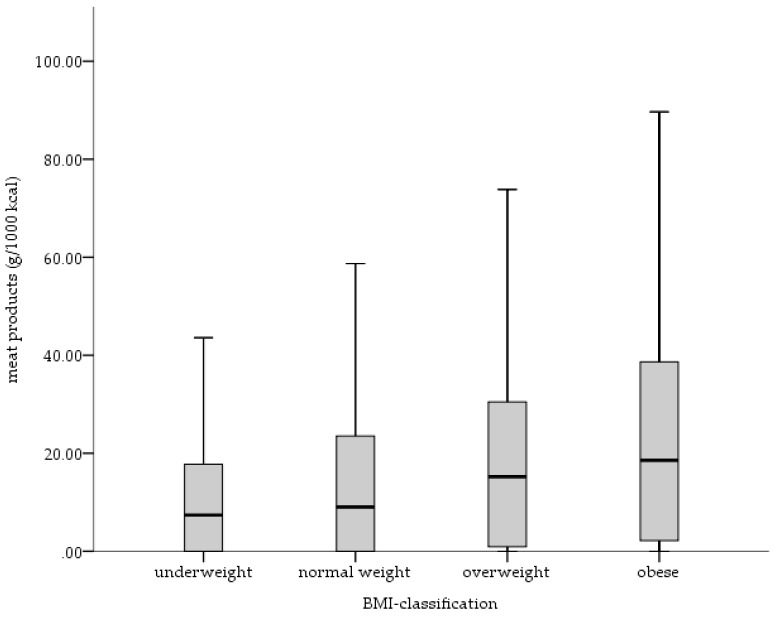
Daily intake of meat products (g/1000 kcal) by BMI-groups.

Overweight and obese persons consumed significantly higher amounts of meat products (z = −5.054, *p* = 0.01; z = −5.150, *p* = 0.01) than normal weight persons (Figure 3). Considering the other food groups, no significant differences were observed.

### 3.5. Predictors of Salt Intake

A generalized linear model was used to study the association between salt intake and potential predictors. As a dependent variable salt intake in mg/day and as factors sex, BMI, energy intake and MMDS score were included into the model. Due to missing data, other potential predictors were not considered. The results showed significant associations regarding sex, BMI and energy intake (Table 4). Due to inhomogeneous distribution, an additional analysis was performed with logarithmically transformed salt intake data, which, however, only minimally modified the results (data not shown).

## 4. Discussion

The data show a mean dietary salt intake in Austrian adults of 5.6 (median: 5.0; IQ range: 3.1) g/day, which is lower than the average intake in adults of most European countries (7–13 g/day) [16]. Considering that dietary salt intake estimated by nutrition surveys may be underreported by an average of 29 to 41% [23] and that the presented data do not fully cover the discretionary salt added at the table or during cooking, the actual total salt intake is underestimated. Assuming that the average urinary sodium excretion is approximately 1.4 to 1.7 times higher than the estimated sodium intake by nutrition surveys [23], the median salt intake in our study would vary from 7.0 (IQ range: 4.4) to 8.5 (IQ range: 5.3) g/day which is similar to Germany (8.4–10 g/day) [45], Italy (9 g/day) [46] and Greece (10.7 g/day) [47].

However, the aim of the presented analyses was not to estimate the bias of nutrition surveys for evaluating the actual salt intake but to assess socio-demographic correlates of dietary salt intake and dietary sources of salt consumption rather than the absolute quantity of salt intake.

### 4.1. Socio-Demographic Correlates of Dietary Salt Intake

Although, biological and socio-demographic correlates of high dietary salt intake are hardly modifiable and therefore not very useful in regard to public health interventions, they can give an insight into specific target populations.

The presented data show, even adjustment for energy intake, that dietary salt intake is higher in men than in women, which may be attributable not only to the actual amount of foods consumed but to the increased intake of food rich in salt. These findings are in line with other studies [48,49,50]. The intake of meat products, for example—one main dietary source of salt—is significantly higher in males than in females, even after adjusting for energy intake. The intake of cheese is significantly lower in males than in females but meat products are a bigger contributor to salt intake than cheese. This is particularly important because a meta-analysis on the relation of food groups with the risk of hypertension observed a positive association between the risk of hypertension and red meat intake as well as processed-meat consumption. With regard to dairy intake an inverse association was observed [51].

Additionally, body weight, body height, body mass index and waist circumference are positively associated with dietary salt intake. Consequently, dietary salt intake is higher in obese than in normal weight persons, even after adjustment for energy intake. The association of an increased risk of obesity with high dietary salt intake has been shown in recent studies [52]. One potential cause for the fact that obese persons consume more salt may be the excessive consumption of processed foods that are relatively high caloric, high in fat and high in salt, respectively. Furthermore, salty foods are more palatable and encourage people to consume greater quantities of these foods [8,9]. Our analyses show that after adjustment for energy intake overweight and obese persons consume significantly higher amounts of meat products—which are relatively high caloric and high in fat—than normal weight persons.

However, recent studies suggest that there may be a link between dietary salt intake and obesity independent of energy intake [52]. Although the mechanism for such a link is not clear, there are some plausibly explanatory approaches. An experimental study in rats showed that rats with a higher salt intake had higher plasma leptin levels as well as excessive accumulation of white adipose fat compared to rats with lower salt intake. Investigations on effects of sodium consumption on insulin and carbohydrate metabolism showed controversial results. While some studies reported a strong relationship between high salt intake and insulin resistance in human and animal models, others showed a hyperinsulinemic response to an oral glucose overload and an accentuated impairment in whole-body insulin sensitivity related to salt restriction. Fonseca-Alaniz et al. reported higher insulin sensitivity and responsiveness in periepididymal (representative of visceral) adipocytes potentially responsible for the rise of adipocyte volume and adipose mass. Adipose mass increase cloud be due to elevated glucose uptake and triacylglycerol synthesis following high insulin serum levels and increased activity of some lipogenic enzymes (glucose-6-phosphate dehydrogenase and malic enzyme) in high salt fed animals [53,54]. Epidemiological studies showed similar results namely the positive association between dietary salt intake and subcutaneous abdominal adipose tissue, independent of energy intake [55]. Summarizing, high salt intake may contribute to a greater deposition of fat suggesting that in some way sodium alters body fat metabolism but further studies need to be performed to gain more insights into the mechanisms.

Consistent with some [46,56] but not all [47,57], previous findings, our data show that persons who were barely adherent to the modified Mediterranean Diet, had higher dietary salt intakes than those who were medium or highly adherent, with, however, the association being only by trend in the regression model. On the one hand this can be explained by the fact, that an unhealthier lifestyle is related to a higher energy intake and therefore a higher dietary salt intake. On the other hand, the majority of daily salt intake is highly attributable to processed foods, traditionally underrepresented in the Mediterranean Diet.

Many studies have evaluated the relationship between salt intake and socio-economic status (SES), with contrary results. Although some studies showed higher salt intakes by groups with a lower SES [46,58], other studies did not find such a relationship [59] and are in line with our findings.

### 4.2. Contribution of Foods to Dietary Salt Intake

The main contributor to salt intake were condiments including table salt used for cooking. The second largest contributors were cereals and cereal based products, meat and meat products and dairy products, similar to what has been described in other industrialized populations [49,60]. Especially bread and bakery products, meat products and cheese are the main dietary sources of salt consumption. These results confirm the well-known fact, that in most developed countries, 75% or more of dietary salt intake comes from salt added to processed foods [61].

This underlines the difficulty of an adherence to a low sodium diet, because processed foods take a significant place in a typical Western diet and high-salt foods are ubiquitous. So, individual options to reduce salt intake are limited without abstaining from these foods with the large amounts of salt added during processing for various reasons [62]. Additionally, people’s knowledge of recommended daily intakes, of the relationship between salt and sodium and of the main dietary sources of salt is poor [63,64]. The gap between self-rated and calculated level of salt intake is large [65]. Grimes et al. observed in their cross-sectional study that less than half of 2398 participants were concerned about the amount of salt in food and only 41% believe that their health would be improved by reducing salt in their diet, even so most are aware of harmful effects of excess salt intake on health outcomes like raised blood pressure and coronary heart diseases [64]. However, understanding the key sources of salt in the diet, as well as being aware of own salt intake, is important for enabling persons to judge their current situation and make steps towards positive changes [66].

To achieve the WHO’s 2025 goal of reducing population salt consumption by 30% [67], a reduction of discretionary salt use by information and raising awareness accompanied by the co-operation of the food producers in gradually reducing the salt concentration of processed foods is inevitable. 

Food reformulation is still a great challenge for manufacturers, because a reduction of salt does not only influence the consumer acceptance but also the microbiological safety and quality of the product. The effect of salt reduction on consumer acceptance strongly varies according to product type [68]. Girgis et al. [69] showed that a one-quarter reduction in the sodium content of white bread can be delivered largely unnoticed in the population over a short time period. Conversely, even salt reductions below 20% in some cheeses may lead to a significant decline in consumer acceptance and may influence the microbial growth. To counteract the effect of salt reduction on flavour, salt replacers such as KCl and flavour enhancers are used but despite the potential to retain the consumer acceptance there are some barriers such as production costs and consumer concern regarding the use of artificial flavours [68,70].

However, and additionally, the improvement of the dietary behaviour of the population in line with general dietary recommendations to reduce consumption of meat and meat products and increase consumption of vegetables and fruits will also contribute to a large extend to the overall reduction of salt intake.

### 4.3. Limitations

The methodology used to collect the presented data has some limitations related to memory and bias. 24-h-recalls may lead to misreporting of the frequency or amount of foods, which in turn are significant contributors to the systematic bias of self-reported dietary assessments. Additionally, dietary surveys are often considered unsuitable for estimating population sodium intake as they tend to underestimate intake due to the variability of the salt content in recipes for both, processed and home-cooked foods and the difficulty to quantify discretionary salt use.

Second, the results are limited by the cross-sectional design of the study, which does not allow judgements of causal relations, rather only associations.

## 5. Conclusions

Socio-demographic correlates seem to have a minor impact on salt intake but specific population groups such as males and obese persons, respectively, need to be targeted by public health initiatives in priority. A diet according to the recommendations—reduction of the intake of meat and meat products and increase of the intake of vegetables and fruits—would be beneficial to lower salt intake.

The present analyses indicate the greatest contributors to total dietary salt intake as bread and bakery products, meat products and dairy products. Therefore, a reduction in population salt intake, can only be achieved in tandem with the food producers by the reduction of the amount of salt in processed foods. Furthermore, easy to understand food labelling is needed, as well as health literacy has to be promoted in order that consumer may identify low salt food or meal options.

The data reinforce the need for action on salt reduction to consequently lower the burden of cardiovascular diseases and increase healthy life years.

## Figures and Tables

**Table 1:** Median daily consumption in grams per 1000 kcal per day for the nine components of the Modified Mediterranean Diet Score.

	Men	Women
*n*	736	1282
Vegetables	73	105
Fruits and nuts	35	72
Legumes	0	0
Cereals including potatoes	127	128
Fish	0	0
Meat	54	36
Dairy products	96	128
Ethanol	2.8	0.3
Lipid ratio ^1^	0.4	0.6

^1^ (monounsaturated fatty acids + polyunsaturated fatty acids)/saturated fatty acids.

**Table 2:** Sample characteristics ^1^.

	Men	Women
*n* (%)	736 (36.5)	1282 (63.5)
Age (years) ^2^	40 (21)	37 (21)
BMI (kg/m^2^) ^2^	24.8 (4.5)	22.8 (4.9)
Waist circumference (cm) ^2^	89.7 (12.8)	77.9 (14.2)
Family Affluence Scale (FAS), *n* (%) ^3^		
Low	4 (0.5)	5 (0.4)
Medium	186 (25.3)	378 (29.5)
High	285 (38.7)	507 (39.5)
Not answered	261 (35.5)	392 (30.6)
Education, *n* (%) ^4^		
Low	226 (30.7)	327 (25.5)
Medium	208 (28.3)	386 (30.1)
High	233 (31.7)	458 (35.7)
Not answered	69 (9.4)	111 (8.7)
Smoking status, *n* (%)		
No	530 (72.0)	936 (73.0)
Yes	170 (23.1)	297 (23.2)
Not answered	36 (4.9)	49 (3.8)
Physical activity *n* (%) ^5^		
Recommendation met	419 (56.9)	850 (66.3)
Recommendation not met	98 (13.3)	202 (15.8
Not answered	219 (29.8)	230 (17.9)
Energy intake (kcal/day) ^2^	2386 (900)	1792 (729)

^1^ Misreporters (defined by a ratio between energy intake and estimated basal metabolic rate (BMR) < 0.788 or >2.488 according to Goldberg et al. [37] and Black [38]) were excluded from the analysis; ^2^ Median; IQ range in parentheses; ^3^ FAS was used as a measure of socio-economic status [28]; ^4^ Education: low: ISCED-Level 1–2 (primary education), medium: ISCED-Level 3–4 (secondary education), high: ISCED-Level 5–8 (tertiary education) [42]; ^5^ Recommendation: 150 min of moderate-intensity physical activity per week or 75 min of vigorous-intensity physical activity per week or an equivalent combination of moderate- and vigorous-intensity physical activity per week (≥600 MET-minutes) with at least 10 min duration [43].

**Table 3:** Socio-demographic characteristics and dietary salt intake.

Characteristics	Proportion by Socio-Demographic Characteristics (%)	Salt Intake (g/day)	
		Median	IQ Range
Overall		5.0	3.1
Sex			
Men	36.5	6.1	3.4
Women	63.5	4.6	2.7
BMI-classification			
Underweight	2.8	4.9	2.1
Normal weight	61.7	4.9	2.9
Overweight	24.6	5.2	3.5
Obesity	10.9	5.8	3.8
Family Affluence Scale (FAS)			
Low	0.7	6.0	3.1
Medium	41.3	4.9	2.9
High	58.0	5.0	3.2
Education			
Low	30.1	5.1	3.3
Medium	32.3	4.9	3.0
High	37.6	5.2	3.1
Smoking status			
No	75.8	5.0	3.1
Yes	24.2	5.1	3.2
Physical activity			
Recommendation not met	19.1	5.0	3.2
Recommendation met	80.9	5.0	3.0
Modified Mediterranean Diet Score (MMDS)			
Low adherence	44.8	5.2	3.2
Medium adherence	42.0	4.9	3.1
High adherence	13.2	4.8	2.9

**Table 4:** Associations between salt intake and different predictors.

Parameter	Regression-Coefficient B	Standard Error	95% CI		*p*-Value
			Lower Bound	Upper Bound	
Sex					
Men	1374.908	126.416	1127.138	1622.678	0.000
Women	0 *				
BMI-classification					
Underweight	−833.940	403.468	−1624.723	−43.157	0.039
Normal weight	−553.012	196.434	−940.016	−170.008	0.005
Overweight	−350.954	215.258	−772.852	70.944	0.103
Obesity	0 *				
Energy intake					
Below the recommended energy intake	−1729.149	119.809	−1963.970	−1494.329	0.000
Above the recommended energy intake	0 *				
Modified Mediterranean Diet Score (MMDS)					
Low adherence	322.207	186.669	−43.657	688.071	0.084
Medium adherence	254.975	186.549	−110.654	620.603	0.172
High adherence	0 *				

* reference group; age was included as a metric covariate into the model.
